# P-779. Antibiotic Treatment Selection and Failure by Race and Ethnicity for Uncomplicated Urinary Tract Infection in US Female Patients

**DOI:** 10.1093/ofid/ofaf695.990

**Published:** 2026-01-11

**Authors:** Jacinda C Abdul-Mutakabbir, Seth Kuranz, Virginia Noxon-Wood, Karl M Kilgore, Meghan E Luck, Jeffrey J Ellis

**Affiliations:** Skaggs School of Pharmacy and Pharmaceutical Sciences, University of California, San Diego, La Jolla, CA, United States, San Diego, La Jolla, CA; Inovalon, Bowie, MD, United States, Bowie, Maryland; Inovalon, Bowie, MD, United States, Bowie, Maryland; Inovalon, Bowie, MD, United States, Bowie, Maryland; GSK, Brattleboro, VT; GSK, Brattleboro, VT

## Abstract

**Background:**

Limited studies have explored race/ethnicity (R/E) treatment (Tx) differences in uncomplicated urinary tract infection (uUTI). This study describes oral antibiotic (ABX) Tx patterns and oral ABX-specific Tx failure (TF) rates by R/E in female patients (pts) with uUTI.

**Figure:**
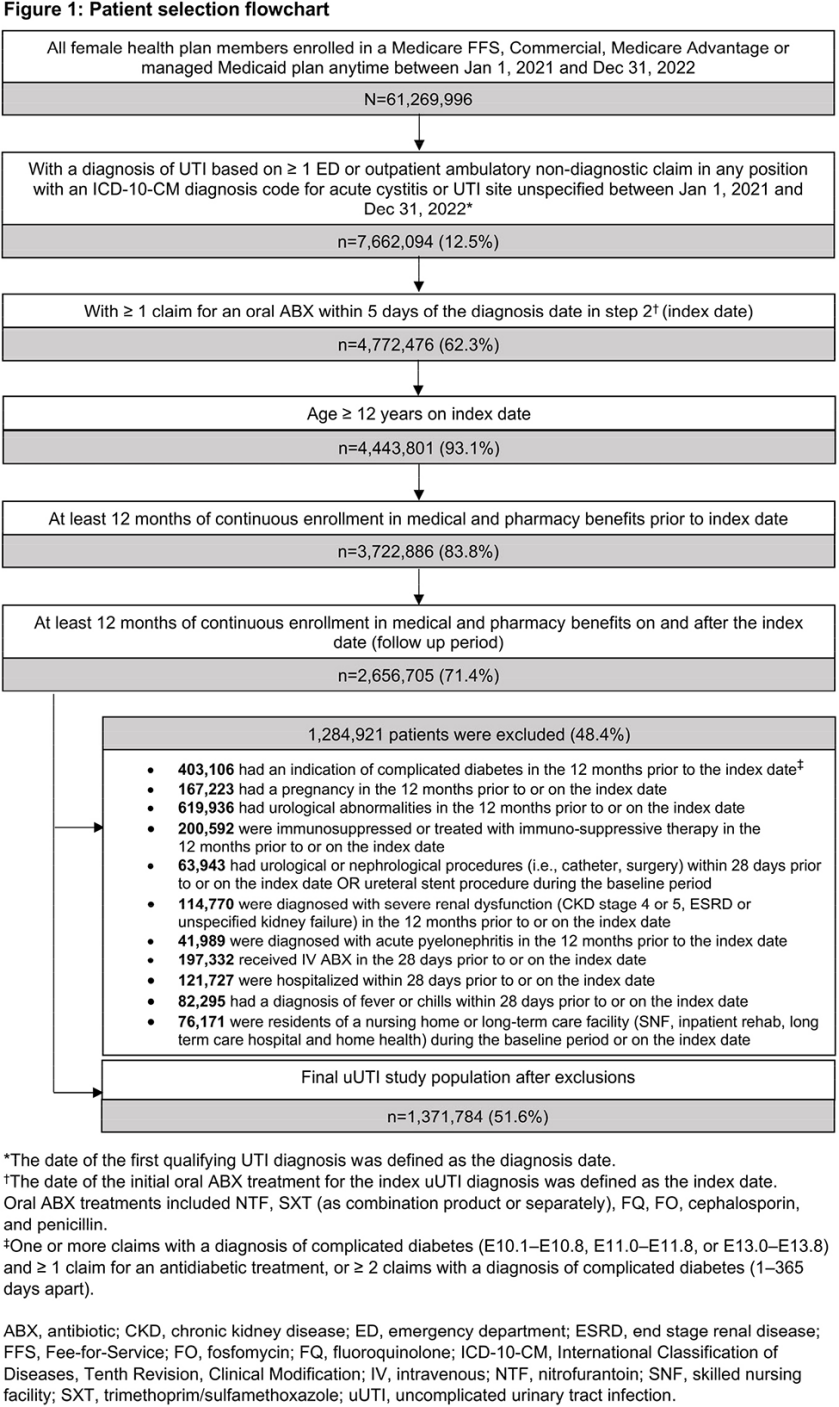

**Figure:**
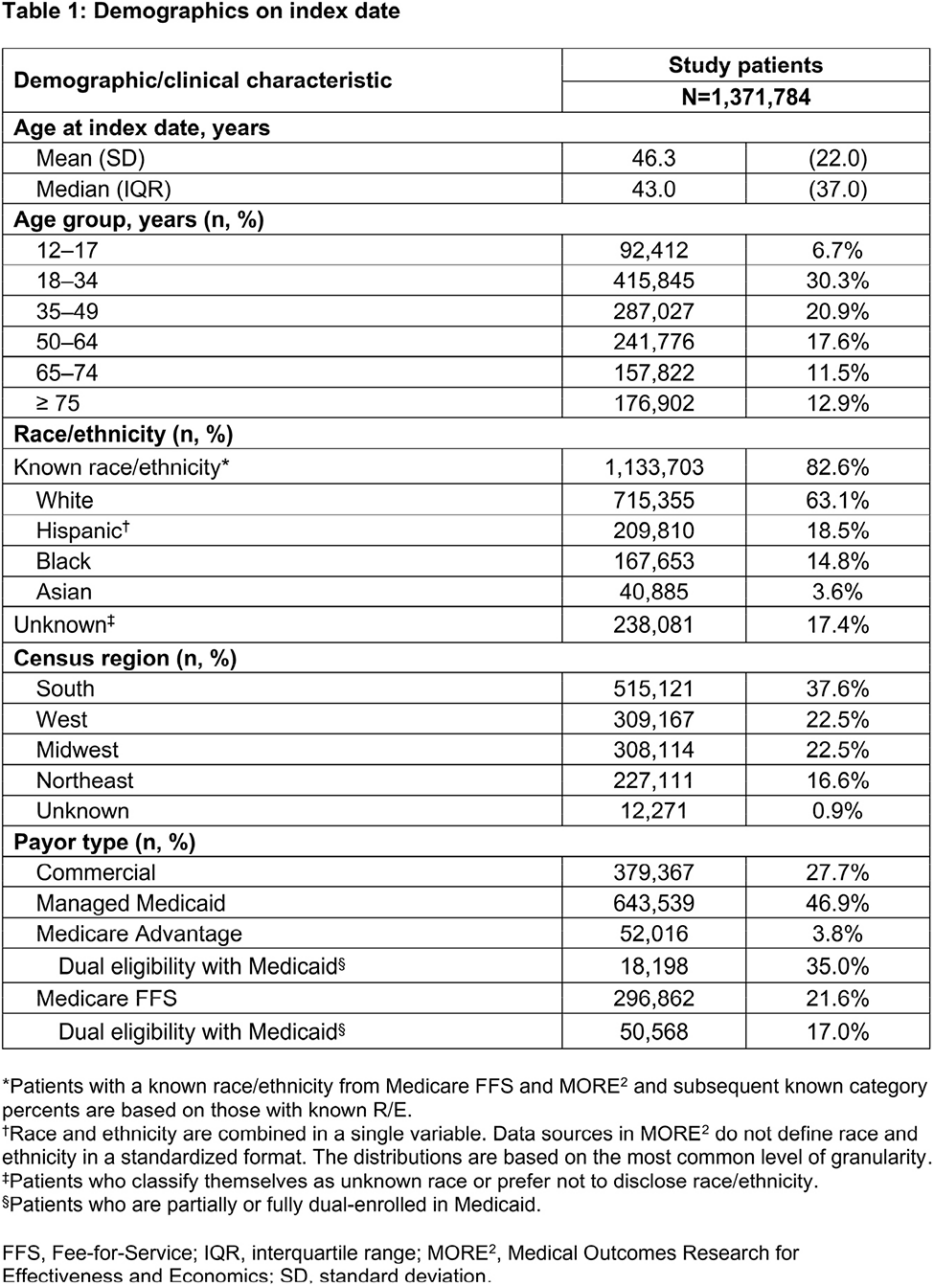

**Methods:**

Females treated in the outpatient setting, aged ≥ 12 years with a uUTI diagnosis (Dx) between Jan 2021 and Dec 2022, were assessed using Inovalon’s Medical Outcomes Research for Effectiveness and Economics (MORE^2^) Registry (commercial, Medicare Advantage, and managed Medicaid lives) and Centers for Medicare & Medicaid Services-sourced Medicare Fee-for-Service (FFS) Research Identifiable Files (all FFS beneficiaries) (Figure 1). Pts with complicated UTI were excluded. TF was defined as having a second oral ABX, intravenous ABX, or an inpatient or emergency department visit with a primary Dx of UTI ≤ 28 days after the index date (first oral ABX claim ± 5 days of uUTI Dx). Due to disparate commercial and government data sources, R/E categories were standardized to the following lowest possible level of granularity: Asian (A), Black (B), Hispanic (H), White (W), and Unknown (U). Data were presented using descriptive statistics.

**Figure:**
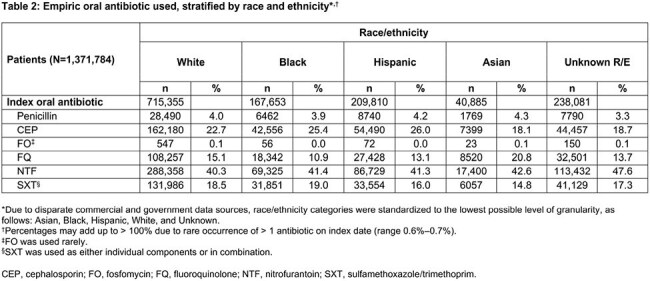

**Figure:**
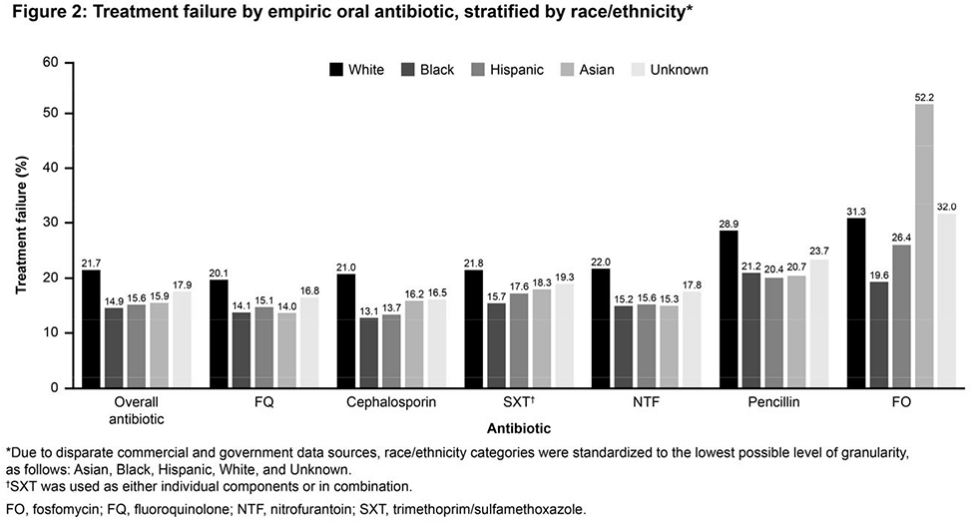

**Results:**

Overall, 1,371,784 female pts with uUTI were included. Most pts (82.6%) had a known R/E identity: 63.1% W; 18.5% H; 17.4% U; 14.8% B; 3.6% A (Table 1). Nitrofurantoin was the most common oral ABX across all R/Es (range, 40.3%–47.6%; Table 2). TF was highest in W (21.7%) and lowest in B (14.9%; Figure 2) pts. Cephalosporin (CEP) use was highest in H (26.0%) and B (25.4%) pts. Fluoroquinolone (FQ) use was highest in A (20.8%) and lowest in B (10.9%) pts. TF was highest among fosfomycin users for each R/E (range 26.4%–52.2%), except for B (19.6%), with A most represented among TF rates (52.2%). TF was lowest among CEP users for B (13.1%), H (13.7%), and U (16.5%) and among FQ users for W (20.1%) and A (14.0%).

**Conclusion:**

A notable proportion of female pts with uUTI experience TF with current oral ABX. Differences in prescribing patterns and TF occurrence between and within R/E were found in this study. Further investigation is needed to determine the impact of clinical factors, access disparities, and other social determinants of health on TF in female pts diagnosed with uUTI.

Funding: GSK study 222864.

**Disclosures:**

Jacinda C. Abdul-Mutakabbir, PharmD, MPH, CSL Seqirus: Advisor/Consultant|CSL Seqirus: Honoraria|GSK: Advisor/Consultant|Shionogi: Advisor/Consultant|Shionogi: Honoraria Seth Kuranz, PhD, Inovalon: Employee of Inovalon, a consulting company that received funding from GSK to conduct this study. Virginia Noxon-Wood, PhD, Inovalon: Employee of Inovalon, a consulting company that received funding from GSK to conduct this study. Karl M. Kilgore, PhD, Karl M. Kilgore: Employee of Inovalon, a consulting company that received funding from GSK to conduct this study. Meghan E. Luck, PharmD, GSK: Employee|GSK: Stocks/Bonds (Public Company) Jeffrey J. Ellis, PharmD, MS, GSK: Employee|GSK: Stocks/Bonds (Public Company)

